# Small Pox—Vaccination and Revaccination—Their Present Position

**Published:** 1851-01

**Authors:** Theodore S. Bell

**Affiliations:** Louisville, Ky.


Art. IV.—*Small Pox—Vaccination and Revaccination—their present position.* By Theodore S. Bell, M. D., of Louisville, Ky.

When Europe was a prey to that direful scourge—Small Pox, which annually immolated 400,000 persons upon its horrid altars, and broke down the constitutions, and disfigured for life the vast multitudes whose cases were not fatal, if any one had appeared in the midst of the pestilence, and proclaimed that a discovery would soon be made that would disarm this plague of its power; that would restrict its ravages within narrow limits; that would so far protect nations from its violence that kingdoms devastated at the rate of 15 and 20,000 per annum, should pass from thirteen to twenty years without having a case of the disease; that with means so mild that the sucking infant, the delicate female, and those who were decrepit from age, might enjoy its beneficent powers without danger, injury, or suffering; that the immunity might be so perfect that a new born infant, whose mother might be covered with the eruption, could draw nourishment from the mother without imbibing contagion, and that vast numbers of persons thus protected, would be free from any danger of taking the disease, no matter how much they might nurse their friends, nor what the exposure might be, the promise would have been looked upon as entirely too visionary in its character ever to be realized, too beneficent ever to be hoped for, and none would have credited it. From the "Indus to the Pole," in all the varieties of the human race, under all the modifications of climate, unrestricted by zones, disregarding all those limits that other diseases respect, this terrific scourge held its relentless course, strewing its path with unnumbered corses, and leaving monuments of its prevalence nearly all over the world. Every tribe on earth that has domestic animals once showed the footprints of the scourge of the human race.

In the midst of the disastrous visitations of small pox, a distinguished English lady, somewhat renowned in literature, announced in 1717, that the Turks had a mode among them by which the disease was greatly mitigated and the mortality very much lessened. Lady Mary Wortley Montague, the lady referred to, said: "The small pox, so generally fatal among us, is here entirely harmless, by the invention of *engrafting*, which is the term they give it. Every year thousands undergo it, and the French embassador observes pleasantly, that they take small pox here by way of diversion as they take the waters in other countries." But it was not so pleasant and harmless as Lady Mary described it. The Turkish invention carried the disease to the doors of thousands whom it would not have reached on its wings. But even the dangerous benefits of that practice were eagerly sought after, so great was the terror in which mankind held the scourge. Its disastrous results in many cases, were forgotten in its benefits in others, and the practice of INOCULATION gradually came into vogue. While it presented strong claims in some points, there were debentures connected with it, that almost counterbalanced its good offices, and made it a matter of reasonable doubt whether it were a curse or a blessing. However beneficial inoculation might be to the individual, he had contracted a contagious disease that often spread disaster in a "natural way." And in all regions of the earth there are places that see the disease only at intervals of time, and thus, unless the fires of pestilence were kept perpetually burning in their midst, there was risk that the inoculating principle would be lost, and the natural contagion would, in a sudden irruption, find multitudes of the young unprotected. Nor was the inoculated small pox always that safe and mild disease that its early advocates represented it to be. It often proved fatal. And it was impossible that confidence could be founded on a method attended with dangers that menaced society; that was not available and safe at all times, that often extended ravages it was intended to prevent, that fell into improper hands for distribution among the young, and was frequently severe and sometimes fatal. For these reasons, instead of restricting the epidemic severities of small pox, inoculation tended to promote them, and often created them. Bills of mortality are the proper test to apply here, to ascertain the results of the practice and they teach that prior to the introduction of inoculation, seventy-two in a thousand cases died, while forty-two years after, the proportion was found to be eighty-nine in a thousand. And though inoculation diminished individual suffering, let the fact be repeated, and fastened upon the memory, that it extended the ravages of the disease by furnishing contagious matter. Inoculation also awakened, developed and often created scrofulous disease, and thereby became a species of engrafting, that was scarcely in harmony with the preservation of the public health. Some years ago I prepared an essay for popular use, on the ravages of small pox, and shall use a portion of the facts then gathered, for the present description.

There is no kind of reason to doubt that small pox was among the early diseases that afflicted humanity. Its independence of climate, position or habits of men; its remarkable aptitude for attacking the human constitution; the pastoral habits of the early tribes, and the universality of its ravages within the period of reeorded history, render the suggestion of its early appearance upon the earth, after man's creation, a highly probable fact. It is or was the universal heir-loom of the human race; the moment that ushered a human being into existence constituted him a mark for this plague, and it hung over him through life, ofen sparing the blow until age had withered the vital powers, and then hurling into the grave those who were tottering on its brink. The earth long groaned under small pox as the scourge of the human race. The first recognizable record of it is, probably, that found in the History of the Peloponnesian war, by Thucydides. No well informed medical man can read the description given by that author of what is called "the Plague of Athens" and imagine that he is reading of what is denominated Oriental Plague. There is scarcely a trace of similarity in the symptoms of the two diseases. And there is certainly no essential feature of small pox that is not mentioned by Thucydides. That pestilence often broke out in walled cities, carrying sorrow, desolation and death among all classes, ages and conditions. It swept off thirty thousand inhabitants in a single year, within the confines of one city. It often attacked immense beseiging armies, and they melted away before it, like frosts before the morning sun. In Prussia, the bills of mortality show that the loss in that kingdom, per annum, when small pox raged in it, was often over fifteen thousand, and rarely ever much less. In Great Britain, the annual loss was from forty-five to fifty thousand persons. The earth stood aghast at the unrelenting scourge, against which science furnished no protection, quarantines no succor, medical hygiene no hope. The capital of Thibet was entirely deserted by its inhabitants for three years on account of the pestilence. It swept off two millions of people in Russia in one year, and three and a half millions in Mexico in a short space of time after it commenced its devastations. About 8 or 9 per cent. of the human race, it was estimated, was swept off by small pox, and an accurate calculator has estimated that fifteen millions of human beings fell victims to it every twenty-five years. In spite of the insular position of England, and in defiance of all her intellectual power, small pox was imported into the island one hundred times in seven years, and in the year 1800, it was imported into England twenty times by the channel fleet alone. These imperfect sketches of the habitudes of the great scourge, sketches of partial spots, are pictures of almost all places on earth. The only blossom that flowered amidst the solitudes of this desolated waste, and in this vast field of death, was found in the consolatory fact that those who lived through one attack, were tolerably exempt from a second.

But while this dark pall hung over the nations of the earth, the power of mind was about to be exerted in disrobing the pestilence of much of its horror. The man and the occasion had come, and the lofty, beneficent and glorious mission of philanthropy could not have fallen into better hands than those of Dr. Edward Jenner. He is entitled to honor for the noble use he made of his rare and wonderful powers, and the medical profession may not only point to him as one of the greatest benefactors of the human race, but as one of the chief dignitaries of humanity, and ornaments of the medical profession. His name and his character will be cherished as long as the human heart beats responsive to a noble impulse.

For a great length of time before the days of Jenner, an idea prevailed that some of the milkers of cows were not exposed to the invasion of small pox. Why they, as a class, should be exempt from a disease which claimed possession of every other human being, seems never to have awakened inquiry in the mind of any medical man, until it aroused the attention of Jenner. Long after he had commenced his investigations of the prophylactic powers of cow pox, he found it impossible to awaken the interest of his medical brethren on the subject. Many of them were on terms of the most intimate intercourse, both professionally and convivially, with Jenner; he was a member of two clubs, in each of which there were medical gentlemen, but they looked upon the stories of the milkers, as they did upon Mother Goose's Melodies, or the records of Jack the Giant Killer. Jenner's medical brethren gave him neither co-operation nor countenance. There was one notable exception to this professional apathy, and it is by no means the least of the pleasant memories and noble honors that are associated with the name of John Hunter, that he warmed and cheered Jenner on his path of discovery. The mind of John Hunter was always awake to every thing that concerned the well being of man, and he urged his highly prized pupil onward in his investigations.

As early as 1770 Jenner communicated to John Hunter the common opinion entertained among milkers, of the power of an inflammatory disease, derived from the cow, to exert a prophylactic influence upon particular persons, by which they were preserved from small pox. The idea had often been heard by physicians, but they made no effort to investigate the statement, or to make any practical use of it. This is a memorable instance which teaches that popular observation is not always false, and should not be rejected without examination. Dr. Davis, of London, forty years after the "Inquiry" of Jenner was published, stated during a debate in a medical society, of the Metropolis, that he remembered an instance in 1798, when he had been ordered to a distant part of England, for the purpose of inoculating a British Regiment, that there were two of the soldiers who refused to be inoculated, alleging that they had been milkers, and had taken a disease from the cows, which protected them from small pox. And while the small pox was in the regiment, one of the men voluntarily slept with persons who had it, and he did not take the disease. The other, although he affirmed he was safe, was inoculated thirteen times by Dr. Davis, and proved his immunity by failing to take small pox. These striking and extraordinary facts made no impression of any value upon the mind of Dr. Davis, and he neither reasoned upon the phenomena, nor attempted to follow them up. It was, perhaps, fortunate for the world, that the mission of delivering the invaluable truths of vaccination fell into the hands of Edward Jenner. His intellectual gifts and manly virtues were precisely adapted to the great and important subject that was destined to influence all the future of the human race. He was an earnest seeker after truth, patient, careful and prudent in the collection of facts, enthusiastic and firm in maintaining them. He was not the creature of impulse; no wild visionary, no reckless dealer in unproved theories, but a true member of that school that feels that nature and observation are worth worlds of fanciful speculation. In perfect keeping with these mental traits, Jenner was singularly free from any thing like selfishness. So full and unreserved was he on the subject that occupied nearly thirty years of his life merely in making investigations, that he might easily have been robbed of the renown of his discovery. But he made it all his own, by the general incredulity of his medical brethren, and his own unfaltering devotion to working out the great pathological and physiological problems before his mind.

In order to give the practical details of this paper their proper fullness, it will be necessary to be very brief in the sketch of the early events of vaccination, although the subject is replete with interest.

It has already been mentioned that Jenner communicated to John Hunter the common belief in the prophylactic power of the vaccine disease, as early as 1770. In unravelling the mysteries connected with it, he devoted twenty-four years of his time, before he vaccinated a human subject. The common opinion, and this influenced Jenner's mind long after he made his discovery, was that it was necessary that the disease should be caught from the cow. Looking back, from the present point of time, the sluggish movements of the truth, and the uncertainty that attended them, look very much like the processes that Charles Lamb ascribes as attendant upon the Chinese discovery of roast pig.

In 1796 Jenner successfully vaccinated the child of his friend Hicks, and afterwards inoculated it with small pox matter, without affecting the child with that disease. In 1798 he published his first work on the subject, and aroused the attention of the public. The services of able coadjutors had been already obtained, but a serious drawback was found in the idea that the lymph for vaccination must come either directly from the cow, or be taken from the arm of the subject on the eighth day of the vaccination. It was often the case that Jenner was in want of vaccine matter for long periods of time. But he had mastered the main points of his great subject, and was armed for the bitter warfare his great discovery brought upon his head.

There is nothing more extraordinary in the history of vaccination than the character of the weapons used against it by a portion of Jenner's medical brethren. It would be but reasonable to suppose that upon a subject of this kind, medical men would have experimented in order to try the new doctrine upon its merits, and that they would have rejoiced in the opportunity offered them for benefiting humanity. But compared to the statements made by some of the profession against vaccination, the scenes described in Munchausen are sober facts, and the language of Billingsgate and St. Giles makes quite as nigh an approach to the refinements of poetry, as some of that employed against Jenner does to truth, dignity and propriety. Three of the physicians referred to attained their bad eminence by the energetic efforts they made for reaching it. Birch, the surgeon of St. Thomas's Hospital, was violent against vaccination. He delivered gratuitous lectures against it, and constantly urged the surgical students to have nothing to do with it. He devoted his life to opposition to vaccination, and flared forth his enmity to it on his tomb-stone. He died in London, and ordered in his will that his tomb-stone should have inscribed on it, that the virtuous being who reposed beneath it "was all his life-time opposed to cow-pocking." His demerits have done for his name and his memory, what his merits would have failed to do. Rowley made himself conspicuous by his ferocity, malignity and falsehood. But Moseley, physician to Chelsea Hospital, excelled even these men in his warfare. He published a book, which proved, contrary to his expectations, to be the sepulchre of his fame. He had handbills published and placarded on the houses of London, in which he affirmed that those who submitted to vaccination were converted into beasts. He gravely affirmed that a lady who had been vaccinated sprouted a pair of horns; he told of cases of vaccinated persons who had their epidermis covered with hair, and who had so far responded to the philosophy of Lord Monboddo as to grow a tail. According to Moseley, there was at least one vaccinated child in Peckham that run on "all fours," bellowed like a cow, and butted like a bull. Some wag, who seems to have gone into vaccination tolerably extensively, undertook to expostulate with Moseley on the horrors of his book. He said:

"Oh Moseley thy book nightly phantasies rousing,
Full oft makes me quake for my heart's dearest treasures;
For fancy in dreams oft presents them all browzing
On commons, just like little Nebuchadnezzars.

*There* nibbling at thistles stand Jem, Joe and Mary,
On their foreheads, O, horrible! crumpled horns bud;
*There* Tom with a tail, and poor William all hairy,
Reclined in a corner, are chewing their cud."

The pulpit too, true to that unity of purpose that characterises its efforts with the gospel, of which it has assumed the management, entered the arena, and sermons were delivered for and against vaccination.

These miserable attempts at opposition, here briefly recorded, had no other effect than to cover their authors with shame and confusion. The beneficent truths of Jenner made rapid progress over the earth. The distant tribes of the world heard that a hand had risen that could stay the plague that had devastated the earth, and which had, hitherto, resisted all other efforts, and in imploring tones begged for the rich boon. No one fact more conclusively establishes the universal dread and horror in which small pox was held, than the celerity with which the news of the discovery of a prophylactic against it, spread through the world. It seemed to move almost with the speed of the electric flash. In an incredibly short space of time, in the circumstances that surrounded the discovery, vaccination was rapidly adopted all over the continent of Europe; zealous, able, and intelligent physicians, animated with the spirit of Jenner, eagerly embarked in the work of extending the new blessing. In the great focus of inoculation, Turkey, vaccination was seized with avidity, and its beneficent gifts were soon given to India, China, and Persia. Throughout the regions of the earth, where the races of men are associated with inferior animals, the protective power of vaccination was eagerly sought after. But a detailed history of these events would extend this essay to an unreasonable length, and the investigation of the results of vaccination, which is most essential to an understanding of the subject, will sufficiently exhibit the diffusion of vaccination.

### Art. I.—*The State of Vaccination during Jenner's life.*

A common opinion is entertained that Dr. Jenner was impressed with the belief that vaccination was a perfect and universal protection against small pox. But he did not entertain any such opinion. In fact, the peculiar view he entertained of the identity of small pox and cow pox, would of itself, have guarded him against such a sweeping conclusion. There were friends of Jenner, active vaccinators, who made the all-engulfing generalization referred to, in which they resembled the boy who ascribed the difference in the length of summer and winter days, to the varying degrees of caloric, but Jenner was not a philosopher of this kind. He believed that human small pox was identical with cow pox, and held that the same laws governed both diseases. He knew of a child in Plymonth, England, who was vaccinated; three years after, during an alarm growing out of the prevalence of small pox, this child was innoculated, and had small pox, and some years after he caught small pox in the natural way. Jenner also knew a lady who had small pox five times. He records also the case of a surgeon, who caught small pox whenever he attended cases of the disease. Jenner was familiar with the case of a boy who was vaccinated in India, and upon his arrival in England was successfully revaccinated. He was afterwards inoculated and took small pox, and sometime after took the disease naturally. Another case was well known to Jenner. It was that of a lady who nursed small pox forty years, was requested to nurse a friend at Bristol, who had small pox, from whom the nurse caught the disease and died with it. She had supposed that she had the disease in infancy.

In view, then, of Jenner's peculiar opinion, that the vaccine disease was a mild form of small pox, the malignity being modified by its passage through the cow; in view of this well known opinion, and of the facts that were familiar to Jenner, it must at once be seen that he was not so ultra on the subject of vaccination as to believe that it was an eternal preventive of small pox. On the contrary, in a letter to Dr. Willan, dated February 23, 1806, Jenner says: "It strikes me that the constitution loses its susceptibility of small pox contagion, *and the capability of producing the disease in its ordinary state,* in proportion to the degree of perfection which the vaccine vesicle has put on in its progress; *and that the small pox, if taken subsequently, is modified accordingly.*" No one contends that Jenner committed no mistake in the great and philanthropic mission he undertook: he undoubtedly committed an error in supposing that vaccination gave as perfect immunity against an attack of small pox, as a primary case of that disease gave against a secondary one. The chief wonder is, now, upon a survey of the subject, after the experience of fifty years, that the zeal of a discoverer did not lead Jenner into more mistakes than he made.

The theory adopted by Dr. Jenner is that the horse is subject to small pox, to which farriers have given the very inexpressive name of grease. It was considered that a high degree of probability attended the opinion that the cow caught the malady from the horse, and there are striking facts that go far towards sustaining that opinion. The facts presented by St. Asaph scarcely admit of any other construction. Mungo Park found a current opinion in Africa that small pox was derived from the camel, and that well-informed and observant traveler coincided in the opinion. There is one form of "grease" in the horse which has all the essential aspects of small pox, and it may be well enough to remark that veterinary surgeons describe three varieties of grease in the horse—the phlegmonous, the strumous, and the variolous. Some years after Jenner's death, Dr. Sonderland, whose scientific position was certified by Hufeland, made experiments to ascertain whether the cow could be infected by exhalations of the contagion of human small pox, and he succeeded. Others have, since that, failed in the experiment. In the 26th volume of the London Medical Gazette, Mr. Ceely published some excellent papers on the various diseases of the cow, which pass under the name of cow pox. No one who undertakes the difficult and hazardous experiment of starting cow pox in the cow, by inoculating her with variolous matter, should do so without studying very carefully the papers of Mr. Ceely. That gentleman succeeded in producing genuine sores in the cow by inoculating her, and thus seemed to sustain Jenner's view of the identity of the two diseases. Dr. Waterhouse, of Massachusetts, states that small pox was given to cows in New England, in consequence of being milked by persons who had small pox. Fiart, in

France, undertook to obtain fresh vaccine virus from the cow, by inoculating her with small pox matter. But he failed, and announced his belief that the disease was unknown in the dairies of France, but soon after this declaration it was found that the disease was raging at Passy, in France. Dr. Baron states that an interval of fifteen years passed, even in the dairy county of Gloucestershire, without a case of the disease among the cows. But *leus bovilla* is often extremely fatal. Thus, in Moidapore, in India, where the experiments of Sonderland were repeated for the purpose of procuring fresh vaccine matter, and in which they failed, the distemper broke out among the cattle naturally, and they died at the rate of from 15 to 20 per cent. The symptoms were very severe; pustules were all over the body, and the ulceration of the mouth and fauces was so extensive that the animals died from inanition. Dr. Alcock believes that a similar state of things in the confluent small pox of human beings is the cause of the great mortality in that disease. But to return: the matter obtained from two of the cows, at Moidapore, that were suffering with the distemper, was used in vaccinating children. The children thus vaccinated were afterwards inoculated without any result. In addition to these facts of cow pox, a multitude of examples might be given in proof of the protective power of the equine matter. Drs. Sacco and De Carro mention examples that should silence all cavilling on that point. It is familiar to the readers of the history of vaccination, that a man, in Paris, caught an eruptive disease in his hands, from dressing the variolous "grease" in the horse, and that numbers of children were vaccinated successfully from his hands. Proof might be piled upon proof on these points, but it is scarcely necessary to pursue it. The question involved in these points is not the one at issue.

The identity of cow or equine pox, with small pox has been questioned. That there is some analogy between the three affections is not a doubtful point, but it is contended that the differences are too many and too great to make a good parallelism. The identity was supposed to consist in the fact that cow pox gave as perfect immunity from an attack of small pox, as small pox itself does. It was inferred that inasmuch as one attack of small pox protected from a second attack in the generality of cases, cow pox would be equally as effectual and general in its effects. Other branches of the phenomena were supposed to be identical. But Dr. Gregory states the differences between the two diseases, in such terms as to almost prove the want of relationship between them. He says: "the VARIOLOUS VESICLE advances with areola from the first; it cannot be perpetuated from man to man in similar states of intensity. Its reception by inoculation is always attended with more or less fever. When received by respiration, the febrile disturbance is often excessive, and may be fatal. However generated it throws off contagious emanations. The infectious principle of small pox is given off from the earliest period of eruption. Fourteen days is the ordinary period of the incubation of the variolous germ. VACCINATION, when first taken from the cow is often severe and produces sometimes an ugly eruption, once taken for small pox. It loses this property by assimilating to the human constitution. The areola is circular, and does not commence until the 8th day. It can be pepetuated from man to man in uniform intensity. It is producible in man by inoculation alone, and not by any combination of common causes. Vaccination may advance and run its perfect course while the system is incubating, and preparing to throw out perfect small pox. In all such cases the period of incubation is free from fever. The lymph of the vaccine vesicle, raised while small pox is thus in the blood, will communicate perfect and uncontaminated cow pock. The small pox thrown out while the system is under the full influence of vaccination is perfect and capable of transmitting genuine small pox to others. The vaccine virus is natural to the cow, or produced by a human vaccination or by small pox inoculation. It does not prevent any cow disease that we know of."

In view of these distinctive features, it would be difficult to establish that identity between the diseases for which Dr. Jenner and his friends contended. Nor is it essential to the value of vaccination that the identity of the diseases shall be established. It is quite sufficient that the appropriate prophylactic powers of vaccination shall be correctly understood and appreciated. If vaccination be equal to its claims, it would make no difference if the antagonism between small pox and cow pox was greater than it is. The theory may be safely surrendered, if the integrity of the practice remains.

### ART. II.—*Failure of Vaccination.*

The violent opposition manifested against vaccination, in its early days caused, as has been shown, much malignant and bitter falsehood, but time began to reveal that the cause itself had salient points, and the bitterness of medical partisanship was not slothful in seizing upon every thing of the kind, and magnifying them. The facts mentioned by Van Sweiten, Heberden, Dimsdale and Hufeland, that mongrel exanthematous diseases, resembling small pox but distinct from it, often prevailed, seem to have been forgotten, and every anomalous event of this kind that happened after the introduction of vaccination, was eagerly seized upon to prejudice the cause of vaccination. But that cause can scarcely be injured in this way. The immunity of immense numbers of the vaccinated from small pox; the myriads who have passed through life without small pox, since the introduction of vaccination, who, according to all past experience and observation among men, would have been subject to that horrid distemper, constitute a bulwark of protection for the cause of vaccination, against which assault is futile. Dr. James Johnson, editor of the Medico-chirurgical Review, says: "there is one earthly evil to which humanity is liable from the moment of leaving the womb, and even before it—that evil is variola." It was so nearly in the nature of an heir-loom, that fond parents hastened to put their children in possession of it as early as possible, because its visitation was looked upon as inevitable.

The varioloid epidemic, in Scotland, described by Dr. Thompson, first shook the public confidence in the protective power of vaccination. It is not necessary to enter upon its details. The conclusion of Dr. Thompson, after a full survey of the epidemic, is more in unison with the purposes of this paper, and remarkably confirms the observation of Jenner, in his letter to Dr. Willan, already quoted. Dr. Thompson says: "it has been impossible to see the general mildness of the varioloid epidemic in those who had undergone the process of vaccination, and the severity, malignity and fatality of the same disease in the unvaccinated, and not to be convinced of the great and salutary powers of cow pox in modifying the small pox in those who were afterwards affected with this disease. Proofs cannot be imagined more convincing and satisfactory of the efficacy of the practice of vaccination and of the incalculable benefits bestowed upon mankind by its discoverer, than those I have had the pleasure of witnessing." And it was in reference to this varioloid epidemic and kindred occurrences, that the truthful and honest-minded Jenner wrote his last opinion on vaccination, on the back of a letter, a few days before his death. He then declared: "My opinion of vaccination is precisely as it was when I first promulged the discovery. It is not in the least strengthened by any event that has happened, for it could gain no strength; it is not in the least weakened, for if the failures you speak of had not happened, the truth of my assertions respecting these coincidences which occasioned them would not have been made out." It is, now, nearly thirty years since those remarks were penned, and time has accumulated many facts on the whole series of questions involved in vaccination. The reader who may carefully read what follows, will be able to place a proper estimate on the whole subject.

### Art. III.—*Small Pox Visitations.*

The small pox sometimes attacked the fetus, often seized upon the newly born infant, and was looked upon formerly as one of the most certain of the discases that assailed humanity. A case is related of a mother, thirty-one years of age, who was vaccinated when an infant. She was delivered of a male infant, iu 1841, in the eighth month of utero-gestation. The premature labor was believed to be Induced by small pox in the child. It was covered with pustules at their height, in the second day of their progress. The pustules matured on the fifth day, and on the ninth, the child died of confluent small pox. A week previous to the labor, the mother felt ill, had a sharp fever, suffered from heat of stomach and loss of appetite.*

Dr. Mead mentions, in his works, a case of small pox in the fetus, and Dr. Jenner describes two cases, in the Medico-chirurgical Transactions.

Dr. Deneuse, in the *Gazette Medicale,* reports the birth of a child, which had the variolous eruption in the 11th or 12th day of eruption at birth. The mother never had the small pox, had seen no such disease, and was vaccinated in early life.

*Bulletina delle Scienze Mediche di Bologna.

In the statistics, published by Mr. Farr, and which are properly regarded as a model, the following fearful facts are stated: Of 9,762 deaths from small pox, in England and Wales, in 1838 and '39, there were.

| | |
|---|---|
| Under 5 years of age, - - - | 7,340 |
| Between 5 and 15 years of age, - - | 1,668 |
| " 15 and 30 " " " - - | 528 |
| " 30 and 70 " " " - - | 210 |
| Upwards of 70 " " " - - - | 16 |

Thus of the 9,762 deaths from small pox, 9,008 occurred at that period of life, in which vaccination exhibits now, an almost undiminished power. From the statistics of the whole world, it is doubtful whether there would have been ten deaths in the 9,008 that occurred between infancy and the fifteenth year of these victims, if they had been vaccinated. In an experience of nearly twenty years in small pox, one year of which exhibited a small pox epidemic, the writer of this has rarely seen a case of varioloid under the fifteenth year, and never saw a severe case under that age.

It is not a little remarkable that there should have been in England, in 1838, and '39, 25,017 deaths from small pox. Taking 20 per cent. as the ratio of mortality, we have 154,000 as the number of attacks in those two years, among a people who had ample power to protect themselves against most of the attacks. These facts show a shameful negligence on the part of the public authorities, as well as among the people. As an evidence of what can be done by proper enforcement of vaccination, it is only necessary to examine results in England. The extensive prevalence of the disease, and great mortality in 1838 and '39 aroused public attention. In 1840 the attacks amounted to 10,434, the year after the new act of Parliament, enforcing vaccination, and prohibiting inoculation, the number sunk to 6,362, and in 1842, to 2,715.

Austria enforces vaccination very rigidly, and the mortality from small pox in that empire, is very low.

But a better view of the character of small pox visitations may be obtained by looking at the returns of deaths in England, prior to vaccination, and before its general diffusion. The National Vaccine Institution makes the following report of deaths from small pox:

| | | | | | | | |
|---|---|---|---|---|---|---|---|
| From 1790 to 1800— | 1,768— | 92 | deaths | to | each | thousand. |
| " 1800 to 1810— | 1,374— | 73 | " | " | " | " |
| " 1810 to 1820— | 833— | 43 | " | " | " | " |
| " 1820 to 1830— | 713— | 35 | " | " | " | " |
| " 1830 to 1832— | 654— | 26 | " | " | " | " |

It is estimated that before the introduction of vaccination, 6 in every 10 deaths was from small pox, and since vaccination was promulgated, about 3 deaths in every 100 were from small pox.

The accurate statistics of mortality in London, show that before the introduction of vaccination, the average amount of death, from small pox, in the Metropolis was 5000, and the average is now about 300, and that number is believed to be in consequence of the continued practice of inoculation. That practice keeps up the disease and spreads it.

The statistics of Ceylon show the character of small pox visitations. That island was formerly ravaged extensively by the disease. The amount of mortality was 110 in 198 cases.

There is no age exempt from small pox. Mr. Bigges, of Newark, England, mentions an instance, detailed to him by Decandolle, at Geneva, in 1833. Decandolle said, that in the early days of vaccination he was present at the Institute of France, when a report on vaccination was to be received. It was highly favorable to the cause; every body was going to be vaccinated, and all the members were going to be vaccinators. Next to Decandolle sat an aged and dignified Duke, who remarked that he had a word or two to say. He said vaccination was well enough for the generality of people, but he had got along eighty-four years without troubling Dr. Jenner, and he should crave leave to continue to do so. One month after, Decandolle was present at another meeting, and he noticed that the chair next to him was vacant, and upon asking where his aged, dignified and well-esteemed neighbor was, he was informed that the old Duke who had felt so independent of Dr. Jenner's discovery, had died with small pox.

In the preliminary remarks to this essay a general view of the awful character of the visitations of small pox was given. In order to complete the picture, a few incidents may be mentioned, and if the reader's attention is fixed upon the multitude of cases that occurred, similar to the following, he may get a true view of the depth of the dark shades that belong to the picture of small pox visitations. In Newark, England, an infant was inoculated for small pox; the mother caught the disease from it and died, leaving six helpless children. A clergyman reported that he was in the habit of burying two or three children every evening, during small pox visitations.

These are but glances at the great subject—the earth was as thickly studded with evidences of small pox visitations, as the firmament is with stars. Nations quaked with fear, districts were almost depopulated, and large cities were entirely abandoned. Terrific as yellow fever, plague and cholera are, they are small afflictions compared with what small pox once was, until the human mind mastered it, and curbed its power. The only solace connected with the evil was in the fact, that a large number of those who lived through one attack, would be secure against a second; but even that consolation was attended with dread—no one of those who had had the disease, knew who might be the subject of a second attack, and as a general rule, those who had passed through one attack found no guaranty in that, that the second would be mild. It may be worth while to look at second attacks of small pox.

### Art. IV.—*Second attacks of Small Pox.*

It was supposed in the early days of vaccination that second attacks of small pox were quite as common as small pox after vaccination, and no less an authority than M. Serres says this is the truth of the matter now. The question is of difficult solution. The complication of statistics in some points, and their barrenness in others, not only create confusion, but cause inquiry to fade into obscurity just at the critical stage of the investigation. It is easy enough to gather statistics from Prussia, Wirtemberg, Norway, Denmark, and England, that seem to say positively, that the proportion of vaccinated persons who take small pox is much larger than of those who have small pox the second time. But these restricted statistics are delusive, and a cheat upon the understanding. They necessarily leave out of view the vast number of persons over whom vaccination continues to exert a protective influence. For instance: Statistics show that a much larger number of persons have small pox in the early period of life than in old age, but it would be an erroneous conclusion to say that old age is less susceptible to variolous contagion than infancy. The disproportion arises from the fact that the disease has performed its mission among the young, and there are few who reach old age, with a susceptibility to the disease. Imagine 2620 inhabitants of a district; say that 2000 of them had small pox in infancy; four hundred take it in adult life, during an epidemic, and of 20 persons, over seventy years of age, 19 take it during the same epidemic, it is evident that this proportion would prove old age more susceptible than infancy, to variolous contagion. And if in any given district there are ten thousand persons who have been vaccinated, and one hundred persons who have had small pox, a large number of cases of small pox after vaccination would be required, to bring that side of the question of liability on a par with the very limited number of persons exposed to small pox a second time. In all these investigations the number of vaccinated persons who escape small pox and varioloid is an important element in the statistical details, and it is universally omitted. An approach to it in some instances gives a cheering view in favor of vaccination, and a full survey of the whole subject will show how much the world has gained by Jenner's discovery. Here in Louisville many persons have had varioloid and small pox after vaccination, and a few have had second attacks of small pox. But then there were so few that had had first attacks, compared to the multitude of vaccinated persons, that it is probable that the relative proportion in the former is quite equal to that of the latter class.

In the records of small pox that preceded the promulgation of vaccination, there are but few details, and the phenomenon of second attacks was either no phenomenon, or it was unnoticed. A fact respecting Sydenham shows how much uncertainty prevailed even in his time on the subject of small pox. The world owes a great deal to that eminent man, for his accurate and judicious views of the treatment of the disease, but it is evident that he did not feel sure that it was a contagious disease. In the edition of Sydenham's works, published in 1666, he expresses the opinion that variola is contagious, but in the edition of 1685, published four years before his death, the edition which he considered the most perfect, he omitted everything that he regarded speculative, and it is not a little singular that among the omitted speculations is the doctrine of the contagion of small pox.

It is necessary then, in order to reach truthful conclusions, that investigations on the point under consideration shall be made among those records that speak distinctly on the subject.

A singular case is detailed in the Transactions of the College of Physicians, in 1785. Two children, in one family, were inoculated; in each a single pustule formed. Matter was taken from these children and other persons were inoculated with it. All of these had fever and an eruption of pustules. At the proper time the first children were reinoculated, and on the 9th or 10th day a smart fever was developed, and with it, an eruption of variolous pustules.

The *Journal Universel* details the following case: a girl had small pox when she was six years old, and seventeen years after she had an attack of confluent small pox, of which she died in the suppurative stage.

Renart reports a case of distinct small pox followed in six weeks by an attack of the confluent form of the disease, which ended fatally.

Dr. Marshal reports, in the London Lancet, 896 cases of small pox, 10 of which were second attacks, being nearly 2½ per cent. In Wirtemberg, of 624 cases, 93 were second attacks, being 1 in 16. Mohl reports, at Copenhagen, 988 cases, 153 of which were second attacks. 31 of the 153 died.

Mr. Lawrence, of the Royal Military Asylum, Chelsea, reports a number of interesting facts. The total number of children admitted in 32 years, from 5 to 14 years of age, was 6,521, of which, 2,532 had small pox previous to admission; 3,080 had been previously vaccinated; 630 were vaccinated in the institution, and 9 had never had small pox, nor vaccination. Of the total number only 62 had small pox in the Asylum; 26 after reputed small pox, and 24 after reputed vaccination. Here then the number of vaccinated persons predominated over those who had had small pox, and the second attacks in the latter case were more numerous than in the former. The second attacks of small pox were one in nearly 98, or 1. 100 per cent; and small pox after vaccination amounted to one in nearly 129, or 1. 1.25 per cent. Five died of the disease, 3 of whom were supposed to have had small pox before; the other two had had neither small pox nor vaccination before.

In 1818 and '19, during the prevalence of small pox and varioloid in Edinburgh, there were more deaths among those who had small pox before, than among those who had been vaccinated. At Norwich, in 1819, the same facts were observed. If there were a parallelism proved between second attacks of small pox and revaccination, and there is certainly good reason for believing that the parallel holds good, the following facts observed at Wirtemberg are of interest: of 297 persons who were pitted by small pox, 95 took vaccination perfectly; in 76 it was modified, and in 126 produced no effect. These facts seem to show that 32 per cent. of these numbers were liable to second attacks of small pox.

Dr. Gregory thinks that between the ages of 5 and 15, since vaccination was introduced, small pox is a rare disease. Among the unprotected, he thinks the ratio is 1 to 2, and among the protected, 1 to 7. These figures appear to be too low in the first class, and too high in the second. The statistics of other observers vary widely from those of Dr. Gregory. The fact is, Dr. Gregory has usually exhibited, in his tables and controversies on vaccination and small pox, much more of the temper of the partisan, than of the spirit of the philosopher. His treatment of Dr. Baron, on the vaccination report to the Provincial Medical and Surgical Association, in 1837, was disgraceful in the extreme, and was well calculated to bring all that Dr. Gregory says on small pox and vaccination into disrepute. But his tables and statements shall continue to appear in this essay, because my desire is to present every thing of interest that has been said on the various subjects that are discussed in this paper. But to return:

Dr. Schleisner, in his Medical Notes on Iceland, says: "one interval of 34 years occurred between two visitations of small pox, in Iceland, and *many* old persons who had the disease in the first epidemic, had it again in the second."

In the Report of the National Vaccine establishment, to Sir Robert Peel, in 1830, it is stated that small pox had prevailed epidemically, in various parts of England for twelve months, and that 28 well authenticated cases of second attacks of small pox had come to the knowledge of the Institution.

In 1828, in an epidemic small pox in Marseilles, France, of 2,000 cases, 20 were second attacks, and of these 4 died.

In Philadelphia, Pennsylvania, a committee appointed, in 1827, to make a Report on small pox and vaccination, state that of 101 deaths from small pox, 10 were after vaccination, and 9 from second attacks of small pox.

The facts are before the reader for his study and inferences. Those, that are detailed in this portion of the essay, bear upon every thing that a careful search could find on the phenomena of second attacks of small pox, from the present, up to the days of Jenner.

### Art. V.—*The protective power of Vaccination.*

The investigations of this essay have reached much the most pleasing, gratifying and cheering of the subjects that belong to the present inquiry. It is a matter of no small moment to ascertain the true position of the protection afforded by vaccination, against small pox. An old physician of London, Dr. Fordyce, who flourished at the time the public mind was agitated with Jenner's discovery, and the violent opposition it encountered, was asked by a friend at a London coffee house, which Dr. Fordyce often visited for the purpose of drinking Port wine, what he thought of vaccination. "I should be very happy," replied Dr. Fordyce, "to give you my opinion on that subject, fifty years hence." The probationary period desired by Dr. Fordyce has expired, and in looking back upon the events of the past fifty years, in connection with small pox and vaccination, we are prepared now to say whether vaccination has been useful, how far it has redeemed the promises of its early friends, and what measure of confidence should be meted out to it. There are few medical inquiries of greater magnitude. On the subject of the early benefits of vaccination there is no kind of doubt any where, but fears are entertained that it is losing its prophylactic powers, and that it may finally cease to be of any efficacy. Many have indulged the fear that small pox and varioloid are increasing among the vaccinated, because the matter used in vaccinating is getting weak, and the hope is fostered that all the evils are to be cured by a resort to the cow again for vaccine virus. These are illusions, however, that will claim attention under their appropriate head.

There is reason to doubt whether vaccination, when surveyed in its completeness is any more uncertain now than it was originally. The world no longer hears of such terrible visitations of epidemic small pox as formerly devastated the earth. In all communities in which the disease appears, large masses of the people are as exempt from the malady as though it were not in existence, and individuals can be protected now from small pox, with as much cerrainty as at any period of the career of vaccination. When the disease shows itself in any region, its ravages can be checked, and held in abeyance. Vaccinated infants can now suck the mother who has the small pox at the time, with as perfect impunity as at any time since Jenner announced his discovery. When unvaccinated persons have been exposed to variolous contagion, they can be as perfectly secured against the disease, as any persons ever were. And vaccination has just as much power to rid the world of small pox as it ever had. No one of the histories of small pox and varioloid epidemics, records the statement that small pox *commences* its ravages among vaccinated persons. It gets its foothold among the unprotected, and then finds only an occasional victim among the vaccinated. These are the truths that are now to be developed in a series of observations gathered from all parts of the world. I ask the reader's deliberate attention.

In all those regions of the earth that were formerly subject to small pox visitations the good effects of vaccination were prompt, decisive, and recognizable. In Norway, the beneficial results of vaccination were so marked, that there was not a case of small pox in that kingdom for ten years, after the introduction of the prophylactic. So entirely exempt were all classes, from fear of the disease, of which they once stood in awe, that the teachers of medicine had ceased to refer to its existence, in lecturing to their classes. This fact shows how perfectly the kingdom was released from visitations of the pestilence. In Denmark, Sweden, Prussia, Austria, Russia, Italy, Spain, France and England, the results of vaccination were in the highest degree interesting and satisfactory. Jenner lived to hear that 230,000 of the Chinese had received vaccination, and one of his friends pleased him greatly by sending him a pamphlet, in the Chinese language, in which was developed the Jennerean doctrine.

The Royal Vaccine establishment, of England, reports, that in seven years 83,647 persons were vaccinated in

England, and of that number only 2 deaths from small pox were authenticated. The Report says, that of 10,000 persons vaccinated, probably not more than 5 die. It is not a little singular, that the English did not pay more attention to the cause of vaccination. While thousands were perishing annually in the heart of England, under the very eyes of the government, for the want of a little Parliamentary attention, British philanthropy was expending all its sympathies upon the negroes of Brazil, Jamaica, and the southern states of North America.

Dr. Hennen, a military surgeon in Wellington's Peninsular campaigns, bears decided testimony. He says: "Small pox has raged around our camps and barracks, and carried off its victims under our very walls, and even from houses where our detached troops were quartered, while it has left them unmolested."

Mr. Field, surgeon to Christ's Hospital, gives the statement, that of 1,877 children, in that Hospital, all between 7 and 15 years of age, during a period of ten years, there was only one death from small pox.

In 1845, a commission consisting of Magendie, Roux, and Serres, reported, that after three years investigation of the subject, facts showed that the preservative power of vaccination was general in its good results.

Dr. Bosquet give the results of 30 epidemics, between 1816 and 1841. The number of persons unvaccinated, attacked with small pox, was 10,434, of vaccinated persons 5,963. Of the former, 1,682 died, or nearly 17 per cent. Of the latter class, 62 died, or a little more than 1 per cent.

The district of Rezat, in Bavaria, containing a population of 500,000 inhabitants, extirpated small pox so completely, that from 1807 up to 1824, there was not a case of the disease.

In France more than 2½ millions of people were exempt from small pox for 13 years. There was not one victim to the disease, in Philadelphia, in 1812, '13, '14, '15, '20, '21, '22. The report made by the committee, appointed in Philadelphia, to report the facts, in their possession, on the subject of small pox, shows the inestimable value of vaccination. Various other American cities show results in the highest degree satisfactory.

In Holstein, Dr. Luder says: "in 234,959 vaccinated persons, between 1801 and 1822, there was not a case of small pox; up to 1824, only 2 cases. In Denmark, for the same period, out of 447,605 cases of vaccination, there was but one case of small pox.

Dr. Kinnis, in a survey of small pox attacks in Ceylon, bears strong testimony to the beneficial effects of vaccination. In 1819, there were 7,874 cases, and 2,945 of the number died. In 1830, after vaccination was extended partially over some of the districts of the island, there were 896 attacks of small pox, and only 167 deaths. But mark the differences. In the maratime districts vaccination was generally used, and in 1830, in 1,030 attacks, there were 147 deaths. In the Kandyan district, vaccination was little used, and in 198 cases of small pox, 110 died. In 1819 there were 100 attacks for every 10 in 1830, and for every 10 that died in 1830, 176 died in 1819. If vaccination had been universally diffused in Ceylon, in 1819, and 1830, 9,000 lives might have been saved.

The reader has only to apply these facts to all parts of the world, to reach proper conclusions. The features developed in the statements here presented, are in accordance with universal observation, and it is not deemed necessary to burthen this paper with statements that would only confirm the view obtained from those given, without throwing any additional light on the subject. These cases have not been selected to sustain a partisan view, but to show the actual bearings of vaccination. As far as the demands and results of revaccination may be considered as militating against the early claims of the friends of vaccination, those demands and results will exhibit the per contra of the benefits of vaccination.

Before passing, however, from a review of the facts which show the protective power of vaccination over districts of country, among large masses of people, it will not be amiss to look into its influence upon individuals. This examination will have the double power of showing the value of vaccination in individuals, and of preparing the way for a consideration of the present value of the matter used in vaccinating.

### Art. VI.—*Influence of Vaccination upon Individuals.*

Mr. Elwin reports a case where an unvaccinated child had small pox. There were three other children, all young, in the same room with the disease, and they had never been vaccinated. *Seven* days after exposure to the infection, the three children were vaccinated successfully, so far as the vesicles were concerned. Two of the children had eight small pox pustules, and the third, an infant, escaped the disease.

During the epidemic small pox, that has prevailed in Louisville this season, I have seen many examples of vaccination which show that its prophylactic powers have not deteriorated. I will name the characteristics of one example, as an illustration of those of many cases, merely remarking that I have seen nothing that throws even a suspicion upon recent vaccination. It seems to do as well now, as it did when Jenner first announced its benefits. The example to which I have referred as illustrative of many, occurred on Third street, in this city. The oldest of three children in a German family, was attacked with a violent convulsion, accompanied with intense fever. At my first visit, I suspected that the case would turn out to be variolous, and at the end of forty-eight hours, my supicions were justified. The two children, younger than the sick one, slept in the same bed with him, up to the time of eruption, and two days after that was developed, I vaccinated the two children. It was successful with them; revaccination was equally so with the mother, and the father failed to take revaccination, though the attempt was made. All the family, with the exception mentioned, escaped varioloid and small pox. And I saw numerous instances in November and December of 1850, equally as palpable as these in showing the value of vaccination.

Mr. Taylor mentions that the protective influence of vaccination was well manifested at Crickland, England. Among other evidences, he mentions a fact which shows that a common opinion among medical men, that a person who cannot take vaccination, is not in danger of small pox, has its limitations. Mr. Taylor says: "of four children exposed to infection during the epidemic, two took vaccination, and it failed in two; the latter of the two classes had small pox."

I repeat that during the present epidemic of small pox, in Louisville, I have seen nothing to shake my thorough confidence in recent vaccination. Other departments of this subject will develope additional facts on the value of vaccination, and they shall be attended to in their proper places.

There is one point of a highly important character, which should be impressed upon the mind of the practitioner in small pox cases. I allude to *the modifying influence of vaccination*, even when *small pox is in the system.* This is a point that is too much neglected, and the neglect arises often from erroneous views of revaccination. The testimony of the power of vaccination in modifying small pox, even when the constitution has imbibed the contagion, is very conclusive.

Dr. Tardieu mentions a case, which he vaccinated after the small pox eruption appeared upon the skin, and it was materially modified by the vaccination.

Mr. Gardner, at the Mauritius, speaks of the excellent effects of vaccination, in modifying small pox, in those suffering with the disease, and in protecting those who had been exposed to the contagion. Mr. Markwick bears strong testimony on these points.

Mr. Metcalfe says: vaccination may be performed after great exposure, after maturing, the small pox may show itself, but if it does, it will be in an abortive form.

Mr. Marston reports a very interesting case, which appears to say that the child referred to was a candidate for small pox, with purpura, and that its chances for getting the disease were very fair. Mr. Marston is surgeon to the Vaccine and small pox Hospital, London. He vaccinated a child, 4 years old. The day following, the five vaccine vesicles were all of a dark mahogany color. There was hemorrhage from the nares and ears; purpura on the face, neck, trunk and extremities. But the case was mild. With this hemorrhagic tendency, if small pox had been taken in the natural way, this child must have died. Those who wish to see a description of small pox, in its most horrid form, may do so by reading "an abstract of a paper, on small pox, complicated with Purpura Hemorrhagica," read before the Obstetrical Society of Dublin, March, 1850, by Mr. Collis.

The case reported by Mr. Marston, quoted above, is another of those incidents which meet the investigator of small pox and vaccination, that seems to show the identity of the two affections. The tendency of small pox to develope purpura hemorrhagica, and the developement of that state of things in Mr. Marston's case, by vaccination, show a relationship that can scarcely be called accidental.

A correspondent of the Boston Medical and Surgical Journal, in 1849, in speaking of an epidemic small pox in Chester, N. H., says: "of 46 cases, 23 were among the unvaccinated, and 23 among the vaccinated. The latter have been very mild; *some of them merely the symptoms without an eruption*, others more strongly characterized. Of the unvaccinated, many have been very severe; some of them the benign, others the distinct, but a majority the confluent or a combination of the two latter forms." I am at a loss to know what this medical correspondent means by cases of small pox without any eruption. It is a medical solecism which is inexplicable. The only pathognomonic symptom of small pox is the eruption; it is that alone which enables the physician to recognise the existence of the disease, and when it is absent, the disease is absent. If vaccination prevented the eruption of small pox in some of the cases at Chester, it performed its proper mission—it prevented the disease in those cases. An eruptive disease without an eruption is an absurdity. It is the play of Hamlet, with the part of the Prince omitted. Those symptoms that are said to constitute variolous fever without eruption are common to small pox, measles and scarlatina, and when these three diseases are prevailing together, as they often prevail, I should think it would be a matter of some difficulty, for even the facile diagnosis that recognises eruptive diseases without the eruption to tell which form it was deciding upon.

Dr. Lombard says; in the *Gazette Medicale*:—In the department of L'Ariege, where small pox has been prevalent for several years, and recently with much severity, a hundred and twenty vaccinations were practiced in a period of six weeks. Of this number of infants, not one was attacked with small pox, while others died. Even where small pox was developed to a certain extent, vaccination exerted a favorable influence. M. Lombard states that in those persons in whom variola appeared, and who had been vaccinated some time previously, the severity of the disease increased with the length of that interval. M. Lombard concludes in favor of revaccination.

Dr. Schäffer reports in the *Medicinische Zeitung*, that in a Prussian district, the small pox attacked 139 persons in 8 years; 47 of the number had not been vaccinated, 92 had been. Of the unvaccinated 15 died, of the vaccinated one died. 121 persons, who had been vaccinated, nursed the sick, and no one of them caught the disease.

Throughout the epidemic of small pox that has prevailed in Louisville, this season, I have known great numbers of vaccinated persons, expose themselves freely to the contagion of small pox, and the great mass of them escaped as perfectly as though they had been nursing cases of chill and fever. I have seen a larger proportion of persons who had been vaccinated attacked with small pox, this season, than I have ever seen before. But I have also seen a much larger portion escape than at any former time, for I have seen many more expose themselves in this epidemic than at any former period. My observations during this epidemic have strengthened my confidence in vaccination. The per centage of attacks, when the vast number who escaped are considered, is exceedingly low. In fact, different countries present a riddle for solution, in the great differences observed in susceptibility to revaccination, and to small pox after vaccination. In some regions a majority of vaccinated persons can be successfully revaccinated, and in such places, a great susceptibility to small pox and varioloid, after vaccination, manifests itself. In the various States of this Union vaccination exerts a more extensive and enduring power, than in any other part of the world. But more of this, in a more appropriate place.

### Art. VII.—*Vaccine Scars.*

A great many persons are in the habit of pronouncing a judgment on the qualities of an old vaccination, by the character of the scars. This is too important a branch of the subject of this essay, to be passed by without investigation. It is of more than ordinary moment to determine whether the scar affords any means of deciding a former vaccination to be a good or a bad one.

It is very questionable whether any marks are left in the skin by vaccination which give positive indications of the character of the vesicle. A large, irregularly shaped scar may be safely pronounced a defective one, but then the most perfectly formed scars are often proved to be defective, by an invasion of small pox. An American medical professor, Dr. Miller, of Baltimore, announced some years ago, that the infallible marks, were pits in the vaccine cicatrix and grooves in the variolous scar, and he declared that as soon as these marks began to disappear, a susceptibility to small pox was approaching. Time and extended observation have proved the fallacy of these tests.

Mr. Hutchinson, of the Foundling Hospital, reports a number of revaccinations. Eleven cases went as regularly through the second vaccination as though they never had had the disease. Eight of the eleven presented perfect cicatrices from the first vaccination, while in three arms, in which there was no trace of a cicatrix left, although it was well known that they had all been vaccinated, the revaccination failed though tried twice.

In a record of 134 cases of vaccinated persons exposed to small pox, 57 escaped the contagion, and the remaining seventy-four yielded to it. Seven of the whole number had, what are considered perfect marks, and one of these took the disease. Twenty-eight had tolerable marks, and fourteen of these caught the small pox. Of 77 with imperfect marks, 30 escaped the influence of the contagion, and of 22 with no scars, seven escaped.

The physicians of Wirtemberg, who enjoyed excellent opportunities for observation, believe that no safe conclusion can be drawn from the marks of the cicatrix, and, although I once had full faith in those marks, subsequent experience has satisfied me of the correctness of the opinion of the Wirtemberg physicians, and no prudent physician would decide as important a question as the security of an individual against small pox, by indices as uncertain as those of the vaccine cicatrix. I have seen varioloid in persons who had excellent scars.

### Art. VIII.—*Revaccination & Small Pox after Vaccination.*

A bitter controversy raged a few years ago, in England, between the intimate friends of Jenner, and those who felt strong inclination to lower vaccination below its real merits. In this controversy a great many interesting facts were developed, and most thoroughly sifted. Amidst the angry passions of the controversy valuable truths came into existence, and we may now gather useful lessons from them.

The remarkable degrees that have been observed in the susceptibility of individuals to small pox and vaccination are very curious. While there are some persons who seem never to lose the susceptibility, but promptly catch small pox every time they are exposed to it, no matter how often that may be, there are others who never possess the susceptibility. They neither take small pox nor vaccination. Mr. Bigges, of Newark, England, knew 4 persons, of both sexes, in one ward, in that city, who resisted frequent exposures to small pox. They were past middle age, and one of them slept with his child covered with eruptions. Twelve persons, in Newark, have always resisted inoculation, often repeated, and eleven others had equally resisted vaccination. I know several persons of the unsusceptible class in this city, and two of the number are old citizens. But the reader must be guarded against an error here. Those who have a latent susceptibility must not be confounded with those who have none. I have seen cases resist twelve, fourteen, and even twenty attempts at vaccination, but they were finally brought under the influence. An interesting case of this latent susceptibility presented itself this season, in the person of the second daughter of Professor Silliman. Attempts to vaccinate her commenced when she was three weeks old, and were continued without success until this season. These attempts amounted to twenty, spread over between three and four years. I made several efforts to vaccinate this case, and used fresh matter each time. I succeeded finally, and never had a finer vaccination.

A common opinion prevails that where a strong insusceptibility to small pox exists, the insuscepibility to vaccination is equally as strong. There is a good deal of probability in the opinion, but it should not be tried too far. In matters of this kind it is not safe to assume that apparent parallelisms amount to proof of positive identity.

But let us survey the phenomena connected with small pox after vaccination. It is supposed that vaccination loses its power after a certain number of years, but there is no analogy in physiology or pathology, on which such an opinion as this can be based. The phenomena upon which the idea is founded, occur in small pox, but that never wears out. If there are cases, as we know there are, who can be successfully vaccinated two or three times in rapid succession, while others lose all susceptibility after the first vaccination, and if the common practice is to dismiss all children with a single vaccination, must there not, *ex necessitate*, be a large amount of latent susceptibility left among these vaccinated persons? This latent susceptibility is clearly recognised in those persons who resist vaccination a long time, but finally yield to it. It is not all destroyed by one vaccintion, in all cases. Suppose that 700 persons are vaccinated in a district of country; that of this number four hundred have all susceptibility to small pox destroyed by one vaccination; that in others the susceptibility is modified, in others not affected by one vaccination, should we not be justified in looking for the same results we find among the vaccinated? Large masses of them are entirely exempt from all danger of small pox, at once and forever, others have attacks of small pox so mild that it is difficult to recognise the disease, and, from these mild shades, the phenomena pass off into the deepest shadows of confluent portraitures. Precisely similar phenomena present themselves in revaccination. This whole subject requires a great deal of patient observation, and rigid analysis. There are certain irresistible laws in all of Nature's works, which are a riddle to all physical geographers. Such, for instance, as that universal and unchangeable law of the human races, announced by Bouè, in connection with the trends of mountain ranges. All over the earth, where mountain ranges extend from north to south, there is a greater fusion of languages and customs, among the inhabitants, than where the range runs from east to west. Take the mountain range between Hungary and Poland as an example of the first; that between England and Scotland, as the last. In one case there is universal antagonism, in the other, universal tendency to fusion. These differences are independent of altitude, and seem to depend on the direction of the range. But it is as utterly inexplicable, as anything we know of in medicine. The proper elucidation of the small pox susceptibilities, however, would require a great deal more space than I can spare now. I have indicated what I believe to be a true solution of the subject, and it is certainly a safe one, for it leads to proper practice. But in making inferences from the facts and tables, which I am about to submit, I pray the reader to remember that the important element of the immense disparity, in all communities, between the numbers of persons protected against small pox by vaccination, and those by small pox itself, must be kept before the mind, if the object is to learn the truth.

In Marseilles, in 1828, there were 30,000 persons who had been vaccinated. An epidemic small pox appeared, and 2,000 of the vaccinated were attacked with varioloid or small pox. There were 2,000 persons attacked with small pox, and of this number 20 were second attacks, and four of these died.

Of deaths from small pox in Sweden, after vaccination, the following tables are given:

In 1822, 103 persons, all over 15 years of age.

In 1835, 47 persons, between 19 and 35 years of age.

In Copenhagen:

In 1828–'29, 29 persons, all adults but one.

In 1832, 10 persons, all above 22, and not one case even of the disease among the revaccinated.

The relationship between second attacks of small pox and attacks of small pox after vaccination, is too close to be passed over. The following tables, made in Prussia, possess interest.

In the Prussian army, there were second attacks of small pox,

| | | | |
|---|---|---|---|
| in 1834 | 619 | 38 | deaths. |
| 1835 | 259 | 5 | " |
| 1836 | 130 | 9 | " |
| 1837 | 94 | 3 | " |

That there is an approach to parallelism in certain lines of small pox after vaccination, and in second attacks of the disease, all must admit. I do not undertake to say, nor would I wish to be understood as inculcating anything more than that there is a necessity for a close and well conducted observation of these points. The following table may awaken some attention to the subject. It is gathered from different parts of Heim's report, and he seems not to have noticed how singular the numbers look when brought together. The numbers are founded on observations in 100 cases:

| | with success. | modified. | failure. |
|---|---|---|---|
| Vaccination after small pox | 32 | 26 | 42 |
| Revaccination | 34 | 25 | 41 |

If successful revaccination shows that a small pox susceptibility still lingered in these constitutions, the success of vaccination after small pox, shows the same thing.

While on the subject of parallels, another interesting set may as well be mentioned. In another part of this paper, I referred to a case where a person was inoculated, but who failed to take the disease. A single pustule, at the inoculated place was all the sign of the disease, in the subject of the attempt. But matter was taken from this pustule and inserted in the arms of some children, and they took the disease. The children who furnished this small pox matter that gave the disease to others, without having the constitutional affection themselves, were reinoculated with matter obtained from the second set, and in the new trial they took small pox. The analogous case to this, in vaccination, was furnished by Dr. Jenner, and would serve to show, even in the absence of the proof furnished in a former part of this paper, how well Jenner knew the fact that vaccination did not always protect from small pox. In a letter to James Moore, in 1817, Jenner says: "a female had been vaccinated by means of a single puncture—a good vesicle appeared—from this several of her neighbors were vaccinated at different times during its progress. The woman caught the small pox, and had it severely. This excited alarm among those who had received the infection from her; they were all subjected to variolous inoculation, and all resisted it." A singular parallel also, to this is mentioned by Mr. Berry—two children were vaccinated when quite young. Some years after the small pox was violent in the neighborhood, and the mother was so alarmed, that she wished her children inoculated. Her request was granted—a pustule formed on each child without affecting the constitutional health of either. But from these two pustules numbers were inoculated, and they took small pox.

Now these, and various other analogies between small pox and vaccination are not accidental; nor does the fact that the analogies fail in some particulars destroy the truthfulness of those points where they do not fail.

The fact of the varying results of revaccination, in different countries has already been mentioned, and these variations are seen also in different parts of the same country. In some places the following is assumed to be a law: "before it became customary to revaccinate, when small pox entered a family, it went through it, but its progress is now arrested by revaccination." This is not true in this country. I have seen various instances where small pox attacked a member of a large family, and about whom there was no restriction. None of the rest of the family took the disease, nor was anything done in the way of revaccination. Exemption is often claimed as due to revaccination, when it had nothing to do with preventing the disease. If revaccination had been resorted to in the cases just mentioned, their protection would have been ascribed to it, when it is evident that they were as well protected without it as they could have been with it. Nor does successful revaccination prove that the subject of it was in a condition to take small pox, until protected by revaccination. At the Hospital at Heilbruren, two sisters were admitted, one with varioloid. The other sister nursed the sick one, and slept two nights in the same bed with her, and they occupied the same room five weeks. Some time after this, the nursing sister was successfully revaccinated, but it is plain that it had nothing to do with her protection. But sweeping conclusions must not be made from isolated facts—the footstalk is too weak to support too large a head.

In the investigations of revaccination, there is abundant proof that it is the wisest and safest course to submit to the requirements of revaccination. Hecker says that since revaccination has been introduced into Prussia, and attended to, small pox has almost disappeared from the empire. In the same kingdom it was remarked, that during an epidemic small pox, those divisions of the army that were revaccinated escaped, while those which were not taken through the process suffered severely. In Prussia and Wirtemberg, of 91,516 revaccinations, 41,663 were perfectly successful. In Wirtemberg, in 6 departments, in each class of 100 cases of revaccination the averages were 46 successful, 20 modified, and 34 without any effect.

In Prussia, the following results of revaccination were observed:

In 1833—31 per cent. successful.
" 1834—37 " " "
" 1835—39 " " "
" 1886—43 " " "
" 1837—45 " " "

In 1843 an epidemic small pox was arrested by revaccination, and the revaccinated were almost entirely exempt from attacks.

Heim shows conclusively that revaccination, at once arrested the spread of small pox in the garrisons at Ulm, Stutgard, and Ludwigsberg. It was used without any regard to the cicatrix.

Lurot, a French physician at Bischwiller, made a number of interesting experiments in order to ascertain the truth of Heim's conclusions, and they were amply corroborated. Indeed, the facts every where seem to justify a rigid attention to revaccination, at the proper times. Where it is successful it should command some confidence, and where it fails it does no harm. There is one consolatory fact in revaccination—no matter what the theory may be as to the call for it, whether it be from the wearing out of a previous protection, or from the remains of an imperfect vaccination, or from a latent susceptibility that has not been previously destroyed, revaccination is imperative, and the consolation is, that if the lapse of time renews the susceptibility to small pox, it equally renews the susceptibility to revaccination.

In 1842, the French Academy of Sciences offered a prize for the best treatise on the value of vaccination and revaccination. Thirty-five candidates presented themselves, and it was not until 1845 that M. Serres was able to present a report to the Academy. The following portions of the report are valuable:

"Vaccination preserves the human species from variola, but its preservative power is not absolute. Variola itself, either spontaneous, or produced by inoculation, does not preserve absolutely from future attacks, therefore it is not extraordinary that vaccination should not. Thus, Mead mentions having seen three variolous eruptions take place successively on the same woman: the son of Forestus was twice attacked with variola, and Dehaen states that one of his patients was attacked six times by variola with impunity, but died of a seventh invasion of the disease. Although, however, vaccination is *sometimes* powerless to preserve us from variola, it *always* diminishes the gravity of the malady. This property, which Jenner and his first successors did not even suspect, is thoroughly proved by the various facts which have been recently accumulated. In one of the most terrible epidemics of variola that has taken place in Europe since the discovery of vaccination,—that of Marseilles, in 1828,—more than ten thousand persons were attacked. Of these, two thousand only had been vaccinated, and of that number forty-five only died, whereas, one thousand five hundred of the eight thousand who had not been vaccinated were carried off by the pestilence.

"Vaccine matter evidently loses part of its efficacy in passing from arm to arm: it is therefore desirable to renew it as often as possible. A remarkable fact mentioned by one of the competitors, supplies us with a means of renewing it, as it were, at will. A cow was vaccinated with matter taken from a child. Not only did the pustules rise, but they were communicated to other cows, so that the cow pox was observed nearly in its natural state. The pustules were identical in both cases.

"The propriety of revaccination is now fully established. In Germany, the various governments have been induced to pay great attention to revaccination, owing to the circumstance of epidemics of variola having latterly manifested themselves with a severity to which we had become quite unaccustomed since the introduction of vaccination. Revaccination has, consequently, been resorted to on a very extended scale, and has had the effect of arresting the epidemics. Thus, in Wirtemburg, forty-two thousand persons who have been revaccinated, have only presented eight cases of varioloid, whereas, one-third of the cases of variola have latterly occurred on persons who had been vaccinated. It is principally between the ages of fourteen and thirty-five that vaccinated persons are exposed to be attacked by variola. When there is an epidemic, the danger commences earlier, and children of nine years of age may be seized. Prudence, therefore, requires that, under ordinary circumstances, revaccination should be performed at the age of fourteen or fifteen, and four years earlier if within the radius of an epidemic of variola."

The reader who has paid attention to the facts presented in this paper, respecting Jenner's views of vaccination, and the liability to small pox afterwards, cannot fail to see how carelessly M. Serres had read Jenner's works. It will be remembered that Jenner declared that when small pox attacked a vaccinated person, the disease would be modified in its character, the very fact which M. Serres says Jenner did not even suspect. When I examine the subject of the renewal of the vaccine virus, I shall be compelled to dissent from another of the opinions of M. Serres.

### Art. IX.—*Present Value of Vaccination.*

The attentive reader, who has carefully analysed the preceding facts on the subjects of small pox and vaccination, can be at no loss in determining the present value of vaccination. I have no doubt that in the great mass of persons, who are properly vaccinated, vaccination exerts as great prophylactic powers as at any period of its career. It never assumed to be a better protection than small pox itself is, and the strong probabilities are that it is now as useful as it ever was. If the number of chances for attacks of small pox in a community are diminished by vaccination, the number of chances for small pox after vaccination must be increased, and it is unfair to take one element of these statistics, and base an opinion alone on it. Yet this has been constantly done by Dr. Gregory and his followers. Before we can say anything of the comparative per centage of second attacks of small pox, and of small pox after vaccination, we must know the numbers in the community relatively exposed in each class. And another element of calculation leads to constant error. It is necessary to know the circumstances under which the figures were obtained, and I can imagine few worse places for gathering them than military barracks or a small pox hospital. When Sir James McGregor or Dr. Gregory says that of 100 cases of small pox after vaccination, 60 will be mild, 40 confluent or severe, a physician who has seen the effect of congregating numbers of small pox patients together, can understand the extraordinary character of the figures quoted. Such proportions are never seen in private practice. Mild cases of varioloid or small pox may easily be made grave by placing them in a crowd of cases. I have seen many instances of this kind.

In every community the great majority of vaccinated persons may expose themselves to small pox contagion without any risk. Various facts detailed in this paper clearly show that fact. The reader will remember one instance where 121 vaccinated persons nursed cases of small pox and no one of them took it. A private practitioner who sees the numbers of persons who expose themselves to small pox, with impunity, might conclude that vaccination was almost a perfect protection. In private practice too, the number who successfully take revaccination, according to my observation are a mere cypher. But let me not be misunderstood—it should be tried in all exposed cases, for there is no other way by which any can tell who is and who is not safe. The general observation of medical men throughout the world seems to sustain these conclusions: from early life up to about the 10th year, and from that to the 15th year, primary vaccination exerts its full power. Retzius, of Stockholm, thinks that vaccination retains its power about 13 years; that revaccination lasts in full power up to the age of 40, and manifests some decadence towards the fiftieth year. If these statements were universally true, they would show that the revolutions of the system have a great deal to do with the supposed changes in vaccination. I have never seen but one case of varioloid within less than the 11th year of the vaccination, and I think that universal experience sustains Wendt, of Denmark, in his opinion, that time has its influence upon vaccination. His numerous and well arranged tables sustain that view, but I have not the space to spare for them, and the reader is mainly interested in the conclusion. I have seen, in this season, in Louisville, mild attacks of small pox about the 15th year of the patient; in those whose vaccination was 20 years of age, the cases were more severe, while in those whose vaccination was 30 or 40 years old, no modifying power was perceptible. These observations have been constantly sustained this season. Whether it is the relative ages of the patients, or of their vaccinations that makes these differences is not firmly established. In an epidemic small pox, revaccination should be general, without regard to definite years, for they are not fixed as immoveably by nature, in reference to susceptibility to small pox, as they are in the multiplication table.

Dr. Cape, of St. Thomas's Hospital, thinks, that one revaccination, immediately after the age of 15 would be sufficient for the remainder of life.

But in order that a community shall enjoy the full value of vaccination, they should secure a more rigid enforcement of it, and a better attention to it, than usually prevails. I showed the effect of the English Vaccination bill, of 1840, in the rapid decrease of small pox, in London, year after year, immediately subsequent to the passage of the bill. In all parts of England, the good effects were rapidly perceived. Thus, in Stockport, a town often afflicted with small pox before the bill was passed, and containing a population of 10,000 persons, not a case was registered for two years after the vaccination bill passed. *There are more imperfections in the modes of vaccination than in the vaccine virus.* Jenner noticed, very early, that while almost all that he vaccinated were safe, those vaccinated by others were not generally secure. I have been curious in a close investigation of the subsequent fate of Jenner's cases, and have been struck with the fact, that while the friends and enemies of vaccination have given very minute details of all that could be found for and against the cause, from the death of Jenner, in 1823, down to the present time, I have met with the record of the occurrence of small pox, after vaccination, in but one person who had been vaccinated by Jenner, and that vaccination was 23 years before the attack. Governments should enforce vaccination. Its importance cannot be estimated properly by the ignorant, but they should not be permitted to poison the atmosphere of other people. The Registrar for Nottingham, England, states that a woman in his district who had lost a child by small pox, assured him that she would rather lose half a dozen children by it, *than fly in the face of Providence in having one vaccinated.*" She may have many analogues in this country.

The rules prescribed by Jenner are almost unknown to great numbers of vaccinators, and the marks that distinguished a genuine vesicle from a spurious one are very imperfectly known, and when known are seldom attended to. But upon a matter of the momentous importance of vaccination, the strictest attention should be given.

### *Has the Vaccine virus Weakened?*

A great many persons have cut the knotty points connected with small pox after vaccination, by assuming that the vaccine virus now in use is so far removed from the cow, that it is no longer efficacious, and the opinion is entertained by some persons, that a resort to the cow again, would restore the vaccine virus to all its original strength. Such reasons spring from a very imperfect knowledge of the subject, and a few irresistible facts will clear the matter of its errors. There is no reason for believing that the humanised vaccine virus has power in any degree inferior to that used by Jenner. As long as it makes the proper vesicle, and possesses the characteristics pointed out by Jenner, it is equal to all that he ascribed to it. But here is a point that should settle the question:—if an epidemic small pox appears among adults vaccinated forty years ago, it is evident that the matter with which they were vaccinated was nearer the cow, than that used now, by forty years. And what are the facts exhibited among them, and among children vaccinated with matter forty years farther from the cow, than their's was? Those vaccinated with matter the farthest removed from the cow, are all saved, while the adults vaccinated with matter nigher the cow, suffer. The London Vaccine Institution now use matter in its regular transmission through human beings, from that originally furnished by Jenner, and the officers of that establishment declare that the virus has lost none of its power. The matter used in various parts of Europe, is in regular descent from that obtained from Jenner, and such is the fact of that used in this country. As long as it will produce the vaccine disease perfectly, it is equal to that obtained from the cow, for it can do no more. The best judges of vaccination declare that new virus from the cow produces no better vesicles than the virus in common use, and some of these judges were the intimate companions of Jenner. Gregory says forcibly: "recurrence to the cow for fresh lymph is not a measure lightly to be had recourse to, nor should it be advisable until the old and tried stock has obviously degenerated." The world would run the risk of getting spurious lymph from the cow. Griva, of Italy, says he "can see no perceptible difference between the fresh lymph, and that which has been long humanised." In France, eleven vaccine committees, and 170 private practitioners concur in declaring that "the humanised old matter is as good as the new." This matter has been as effectual in Louisville this season, as Jenner's was in Gloucestershire, in 1806. It has afforded perfect protection in all recent vaccinations.

*The proper time for Vaccination.*

Dr. Murray, of the Cape of Good Hope, and Mr. Grantham, think that vaccination is performed too early in life, and the former gentleman quotes Munro in proof of the fact that young children are not very liable to small pox. I know of no good reason for such an opinion—infants that are exposed to the contagion, so far as I have had an opportunity of seeing such cases, are as liable as children of any age. I have vaccinated infants immediately after their birth, before they were dressed, and vaccination seems to do as well with them as with others, and I have seen such cases exposed sixteen years afterwards to small pox, without taking the disease. If there is exposure or liability to it, the age should not be thought of. Mr. Howison, vaccinator to the Royal Dispensary, thinks that from 1 month to 1 year is the proper time, and balmy pleasant weather more favorable, than any other kind.

Mr. Delfraysse, in a memoir submitted to the Academy of Medicine, Paris, Oct. 8th, 1850, undertook to show why vaccination fails. He thinks that vaccination is infallible, where the vesicles are sufficiently numerous to produce a degree of febrile action. He proposes 20 or 30 punctures in different parts of the body, and says that he exposed cases thus vaccinated, to the greatest degree of contagion, without any evil consequences. But he might have done this equally as safely, if he had made but one puncture, for recent vaccination, with one puncture, is an ample protection. But I think that it would be an improvement to vaccinate children, at various intervals, as long as they show any susceptibility to vaccination, for all the susceptibility is not destroyed in all children, by one trial. But the multiplied punctures commended by M. Delfraysse are not quite as harmless as he thinks them.

Mr. Rees, in the London Lancet reports three cases of sloughing from numerous vaccine punctures—some 12 or 15 in each arm. Two of the children died, and the third was saved with great difficulty.

Drs. Gregory and Colles think a child may be safely and properly vaccinated, when two months old. Heim is in favor of waiting twelve months, and not deferring it beyond the third year. But there is no good reason why a physician should bind himself to any of these rules. Nor is there any reason for believing that a vaccine scab can communicate any other disease, than the cow pock, even if taken from children laboring under scarlet fever, measles, hooping cough, scrofula, or anything of the kind. Physicians are often charged with giving scrofula to children by vaccinating them with matter from scrofulous children. It would be as rational to charge any other subsequent event of a child's life, to the same cause.

Beeker thinks that vaccination is less active in summer than in winter. Of this there is no doubt. During the hot dry weather of last July and August, at least 18 of 20 attempts at vaccination, in my hands, failed. As soon as the weather turned cool, at least 18 in 20 succeeded. Robert Annan, of Kinross, Scotland, has recorded similar occurrences in June and July, 1850, in Scotland.

### *Incubation of Vaccination.*

The usual period of this is well known, but great variations are often noticed. The *Journal Universel* mentions a case where four punctures were made; there was no development for two months, the vaccination then did well, and other children were vaccinated from the pustules. Mr. Brachet, of Lyons, mentions a case in which, on the 10th day there was no development; on the 11th revaccination was resorted to, and then the first vaccination commenced and ran through its course. Thesecond pustules dried up immediately. I once vaccinated a child seven different times, each time with fresh matter. The seventh effort was successful, but there was no sign of the disease, until the fourteenth day.

I have thus endeavored to present, from a great multitude of facts on the various branches of small pox, vaccination, and revaccination, such a selection as most clearly and expressively elucidates each portion of the subject. It is only on the basis of a universal survey of a medical subject that a superstructure of sound philosophy can be built. It involves a great amount of labor, but no one should falter in the work on that account. The recent epidemic small pox, in this city, developed great many facts confirmatory of medical observations in other countries, and it is due to the profession that these facts shall be recorded. And this epidemic found so many medical men unprepared on some of the important points that have been established in other parts of the world, that the cause of vaccination, which is the cause of humanity, absolutely required a rigid analysis of its condition. I believe that it has come through the trial triumphantly, and that it is fully entitled to the confidence and gratitude of mankind.

### Art. X.—*Treatment of the Eruption of Small Pox.*

It would scarcely be proper to close this paper without a reference to the *ectrotic* treatment of small pox. The following are the most recent recommendations of reputable practitioners:

Mr. Stewart says that while the eruption is vesicular or papular, if a small opening be made near the base of each vesicle, and the matter squeezed out, so that adhesive inflammation can be brought about, the disease is mitigated, and pitting prevented. Velpeau recommends the use of lunar caustic. Mr. Grantham recommends that the point of each pustule be cut off, and the eruption dressed with calamine. Picton, of New Orleans, speaks highly of the benefits of the exclusion of light. Larrey had great confidence in a dressing of sweet oil. Legrand covered the face with gold leaf. Chomel speaks favorably of mercurial plaster, and Zimmerman, Rosen, Serres, Briquet, Nonat and Coppen all unite in its favor. They prefer what is called *Vigo's plaster*, of which the following is the recipe:

℞. Mercury 95 parts,
Balsam of storax 48 parts,
Wax, rosin, and turpentine aa I6 parts,
Gum ammoniac, betellicum, 5 "
Olibanum, and myrrh
Saffron, aa - - - 3 "
Spirit of Lavander, - - 2 "

The plaster must be spread on something stiff enough to support itself—and must be closely fitted to the skin. Three days is usually long enough for the plaster to remain in distinct, and four days in confluent small pox.

But so far as my experience has enabled me to form a judgment, I prefer covering the face with strong mercurial ointment, with 2 drachms of starch to each ounce. Better effects could not be derived from any application, than I have uniformly seen from this. I have constantly seen beneficial results from its use, night and morning, until the eruptive stage passes off.

M. Piorry strongly urges the use of blisters, and presents strong reasons in favor of the practice.

In both forms of small pox, but especially the confluent, attention cannot be directed too early to the state of the throat and tongue. Various forms of diseased action present themselves at those points, and, if not attended to early, may end fatally. Apthous ulcers, laryngeal, tracheal, and bronchial inflammations are among the formidable affections that meet the physician in these cases. And I am persuaded that Edematous Laryngitis, so well described by Professor Bartlett in a former number of this *Journal*, is frequently a cause of death in confluent small pox. Dr. Fleming, of Dublin, in an able paper, on the diseases of the tongue, in the *Dublin Medical Journal*, has fully described the various affections of the tongue and throat, to which I am alluding. He found them prevailing extensively in an epidemic variola in Dublin. In the first class of these diseases, I have found a gargle of muriatic acid ℈i and honey ℥ij an effectual remedy; the Edematous Laryngitis can be relieved only by an operation.

